# Posttraumatic Stress Disorder Prevalence and Risk of Recurrence in Acute Coronary Syndrome Patients: A Meta-analytic Review

**DOI:** 10.1371/journal.pone.0038915

**Published:** 2012-06-20

**Authors:** Donald Edmondson, Safiya Richardson, Louise Falzon, Karina W. Davidson, Mary Alice Mills, Yuval Neria

**Affiliations:** 1 Center for Behavioral Cardiovascular Health, Columbia University Medical Center, New York, New York, United States of America; 2 College of Physicians and Surgeons, Columbia University Medical Center, New York, New York, United States of America; 3 Veterans Affairs Boston Healthcare System, Boston, Massachusetts, United States of America; 4 New York State Psychiatric Institute, Columbia University Medical Center, New York, New York, United States of America; 5 Department of Psychiatry, College of Physicians and Surgeons, Columbia University, New York, New York, United States of America; 6 Department of Epidemiology, Mailman School of Public Health, Columbia University, New York, New York, United States of America; Institute of Psychiatry at the Federal University of Rio de Janeiro, Brazil

## Abstract

**Background:**

Acute coronary syndromes (ACS; myocardial infarction or unstable angina) can induce posttraumatic stress disorder (PTSD), and ACS-induced PTSD may increase patients’ risk for subsequent cardiac events and mortality. **Objective**: To determine the prevalence of PTSD induced by ACS and to quantify the association between ACS-induced PTSD and adverse clinical outcomes using systematic review and meta-analysis. **Data Sources**: Articles were identified by searching Ovid MEDLINE, PsycINFO, and Scopus, and through manual search of reference lists.

**Methodology/Principal Findings:**

Observational cohort studies that assessed PTSD with specific reference to an ACS event at least 1 month prior. We extracted estimates of the prevalence of ACS-induced PTSD and associations with clinical outcomes, as well as study characteristics. We identified 56 potentially relevant articles, 24 of which met our criteria (N = 2383). Meta-analysis yielded an aggregated prevalence estimate of 12% (95% confidence interval [CI], 9%–16%) for clinically significant symptoms of ACS-induced PTSD in a random effects model. Individual study prevalence estimates varied widely (0%–32%), with significant heterogeneity in estimates explained by the use of a screening instrument (prevalence estimate was 16% [95% CI, 13%–20%] in 16 studies) vs a clinical diagnostic interview (prevalence estimate was 4% [95% CI, 3%–5%] in 8 studies). The aggregated point estimate for the magnitude of the relationship between ACS-induced PTSD and clinical outcomes (ie, mortality and/or ACS recurrence) across the 3 studies that met our criteria (N = 609) suggested a doubling of risk (risk ratio, 2.00; 95% CI, 1.69–2.37) in ACS patients with clinically significant PTSD symptoms relative to patients without PTSD symptoms.

**Conclusions/Significance:**

This meta-analysis suggests that clinically significant PTSD symptoms induced by ACS are moderately prevalent and are associated with increased risk for recurrent cardiac events and mortality. Further tests of the association of ACS-induced PTSD and clinical outcomes are needed.

## Introduction

In recent decades, survival rates after acute coronary syndrome [ACS; ST-segment elevation myocardial infarction (STEMI), non–ST-segment elevation myocardial infarction (NSTEMI) or unstable angina (UA)] have steadily increased [1], [2]. Thus, for the 1.4 million ACS patients discharged annually from US hospitals, as well as the 200,000 in Italy, 136,000 in France, and similar numbers of patients across Europe and throughout the world, quality of life after ACS has become vitally important. Ironically, though the survival benefit conferred by the adoption of new technologies, interventions, and treatment guidelines is a great triumph of modern medicine, it may have also increased the number of patients for whom survival means living with posttraumatic stress disorder (PTSD) due to the ACS event.

In the *Diagnostic and Statistical Manual of Mental Disorders* (Fourth Edition; *DSM-IV*) [3], life-threatening illness is recognized as an event that can elicit PTSD. Posttraumatic stress disorder is an anxiety disorder initiated by an exposure to a traumatic event, such as combat, disaster, or sexual assault, and is characterized by symptoms such as re-experiencing (e.g., intrusive thoughts, nightmares), cognitive or behavioral avoidance of reminders of the event, and physiological hyperarousal. It is associated with abnormal amygdala, prefrontal cortex, and hippocampal function [4] as well as abnormal neuroendocrinologic characteristics [5]. At this stage, however, the prevalence of ACS-induced PTSD is unknown.

It is also unclear whether ACS-induced PTSD, like other psychosocial vulnerabilities such as depression, is associated with worse survival after ACS. However, epidemiological and observational evidence suggests that PTSD due to traumatic events other than ACS is related to increased risk of incident cardiovascular disease [6], [7], and some previous research has found that ACS-induced PTSD also increases patients’ subsequent risk for recurrent cardiac events and mortality [8], [9], [10]. This review was driven by two questions: What is the prevalence of ACS-induced PTSD, and what is the prospective association between ACS-induced PTSD and objective clinical outcomes, specifically, recurrent cardiac events and mortality?

## Methods

### Search Strategy

We sought to identify all studies that reported a valid estimate of the prevalence of ACS-induced PTSD. To be included, studies must have been observational cohorts and must have assessed PTSD with specific reference to an ACS event that had occurred at least 1 month prior to the PTSD assessment. Further, studies must have used a self-report PTSD screening instrument or clinical interview designed or specifically altered to query about only *ACS-induced* PTSD, not PTSD due to any other type of traumatic event. Potentially relevant articles were identified by searching the biomedical electronic databases Ovid MEDLINE, PsycINFO and Scopus. Dates searched were 1948 to July Week 2, 2011, and the search was conducted on July 21, 2011. All relevant subject headings and free-text terms were used to represent PTSD and ACS and the sets of terms were combined with AND. Terms for MEDLINE were: exp Stress Disorders, Traumatic/OR ptsd.tw. OR (post-traumatic OR (post adj traumatic)).tw or posttraumatic.tw. OR acute stress disorder$.tw. OR asd.tw, exp Acute Coronary Syndrome/OR acute coronary.tw. OR acs.tw OR exp Myocardial Infarction/OR myocardial infarct$.tw. OR (mi or ami).tw. OR (heart adj attack$).tw. OR ((post adj mi) or postmi).tw. OR (stemi or nstemi).tw. OR ((preinfarction or unstable) adj angina$).tw. These terms were adapted for the other databases. Additional records were identified by scanning the reference lists of relevant studies and reviews and by employing the Related Articles feature in PubMed and the Cited Reference Search in ISI Web of Science.

### Study Selection

To determine the studies to be assessed further, two authors (S.R., L.F.) independently read the, title or abstract of every record retrieved. All potentially relevant articles were investigated as full text. Where differences in opinion existed, they were resolved by consensus.

### Database Construction and Coding

From studies describing the prevalence of ACS-induced PTSD, we abstracted PTSD rates and characteristics of the study and sample (**Table 1**). Only current cases of *ACS-induced PTSD* due to the index ACS event were included in PTSD prevalence rates. Where multiple publications from the same cohort were identified, we chose the publication with the largest sample size and the earliest post-ACS assessment of PTSD (eg, von Känel et al [10] was included in the PTSD and clinical outcomes analysis but was not included in the prevalence analyses, because the study by Guler et al [11] had a larger sample size that included the von Känel et al. patients and included a gold standard clinical interview for PTSD). One study’s characteristics were uncertain [12], and the study authors could not be contacted to obtain this information, so this study was discarded. The coding of all articles included demographic information (eg, sex, age, and race), sample size, study location (eg, United States, Europe), method of PTSD assessment (eg, clinical interview or questionnaire), and timing of PTSD assessment. From the longitudinal outcome studies, clinical outcomes (cardiac hospital readmission and combined cardiac readmission and all-cause mortality) were abstracted.

**Table 1:** Characteristics of Studies on the Prevalence of ACS-Induced PTSD.

Source, y	PTSD Prevalence, %	PTSD Measure	Clinical Interview,Y/N	Time After ACS Event, mo	Study Location	N	Male, %	Mean Age, y	Includes Unstable Angina,Y/N
Ayers et al,^35^ 2009	16	PDS	N	2	United Kingdom	74	76	62	N
Bennett and Brooke,^36^ 1999	11	PDS	N	9.24	United Kingdom	44	68	62.5	N
Bennett et al,^37^ 2001	8	PDS	N	3	United Kingdom	39	77	59.7^a^	N
Bennett et al,^38^ 2002	16	PDS	N	3	United Kingdom	75	78^a^	60.4^a^	N
Chung et al,^25^ 2007	31	PDS	N	115.8	United Kingdom	120	78	66.85	N
Doerfler et al,^28^ 1994	11	Multiple	N	9	United States	27	100	59.1	N
Doerfler,^26^ 1997	11	PDS	N	1	United States	30	71^a^	58.9	N
Doerfler et al,^27^ 2005	8	PSS-SR	N	4.5	United States	52	69	57.73	N
Edmondson et al,^24^ 2011	11	IES-R	N	1	United States	247	55	60	Y
Ginzburg et al,^18^ 2003	16	PTSD-I	N	7	Israel	116	81	54.95	N
Guler et al,^23^ 2009	4	CAPS	Y	3.25	Switzerland	394	83	61	N
Kutz et al,^39^ 1994	7	SCID	Y	9.6	United Kingdom	27	89	58.2	N
Lukach,^29^ 1996	0	SCID	Y	6	United States	70	69	59.3	N
O’Reilly et al,^40^ 2004	7	SCID	Y	10.7	United Kingdom	28	89	58.2	N
Pedersen et al,^43^ 2003	22	PDS	N	1.25	Denmark	112	71^a^	60	N
Roberge et al,^32^ 2010	4	SCID	Y	1	Canada	393	NR	59.22	N
Rocha et al,^30^ 2008	4	SCID	Y	1.5	United States	25	NR	NR	N
Sheldrick et al,^41^ 2006	24	DTS	N	1.5	United Kingdom	21	NR	NR	N
Shemesh et al,^34^ 2001	10	IES	N	6	Israel	102	79	61.3	N
Shemesh et al,^21^ 2004	20	IES	N	6	Israel	65	79^a^	53^a^	N
Shemesh et al,^33^ 2006	22	IES	N	7.5	Israel	65	90	58	N
Thompson,^31^ 1999	31	ADIS-IV	N	6	United States	26	NR	NR	N
van Driel et al,^44^ 1995	0	SCID	Y	24	Holland	18	56	61.96^a^	N
Wikman et al,^64^ 2008	12	PSS-SR	N	12	United Kingdom	213	77	61	Y

Abbreviations: ACS, acute coronary syndrome; ADIS-IV, Anxiety Disorder Interview Schedule for *DSM-IV* [11]; CAPS, Clinician Administered PTSD Scale [62]; DTS, Davidson Trauma Scale [63]; IES, Impact of Events Scale [64]; IES-R, Impact of Events Scale–Revised [65]; NR, not reported; PDS, Posttraumatic Stress Diagnostic Scale [66]; PSS-SR, PTSD Symptom Scale–Self-Report Version [67]; PTSD, posttraumatic stress disorder; RI, Reaction Index [68]; SCID, Structured Clinical Interview for *DSM-IV* [69];

a Value reported for full sample, including participants who did not complete the study.

### Quantitative Methods

Comprehensive MetaAnalysis (version 2, BioStat Software, Engelwood, NJ) served as the statistical platform for completing all statistical tests and associated graphic results. To summarize the prevalence findings, we computed prevalence point estimates using these formulas:

Logit Event Rate  =  Log [Event Rate/(1 − Event Rate)].

Event Rate Standard Error  =  √(1/(Event Rate · Sample Size)/(1/[(1−Event Rate) · Sample Size]),

We computed 95% confidence intervals (CIs) using this formula:

Lower Limit  =  Logit Event Rate − (1.96 · Logit Event Rate Standard Error).

Upper Limit  =  Logit Event Rate + (1.96 · Logit Event Rate Standard Error).

For meta-analytic tests of ACS-induced PTSD prevalence, in addition to the overall random-effects model, we used sensitivity analyses to assess evidence of moderator effects for the prevalence results across a number of methodological factors. These factors included the method used to assess ACS-induced PTSD, the location of the study (United States vs other), and whether studies explicitly included patients with NSTEMI and UA. Further, meta-regression analysis was used to test the association between study prevalence rates and timing of the PTSD assessment (number of months after ACS), study publication date, and sample age and sex composition. Heterogeneity assessments preceded all meta-analytic tests concerning PTSD prevalence and clinical outcomes. There was statistically significant heterogeneity, so random-effects models were used to estimate and test effects.

We calculated an aggregated point estimate for the risk ratio associated with a positive screen for PTSD on clinical outcome categories including cardiac hospitalization and a combined endpoint of cardiac re-hospitalization and all-cause mortality. Log-transformed risk ratios and 95% CIs were calculated for each study using the reported effect size and estimates of the SE of each effect drawn from data reported in the article. When articles reported multiple models, we selected the model with the highest level of covariate adjustment. For meta-analytic tests of the association of ACS-induced PTSD and clinical outcomes, there were too few studies to test for moderator effects. To address the issue of publication bias, we calculated a fail-safe N.

## Results

### Literature Search

**Figure 1:**
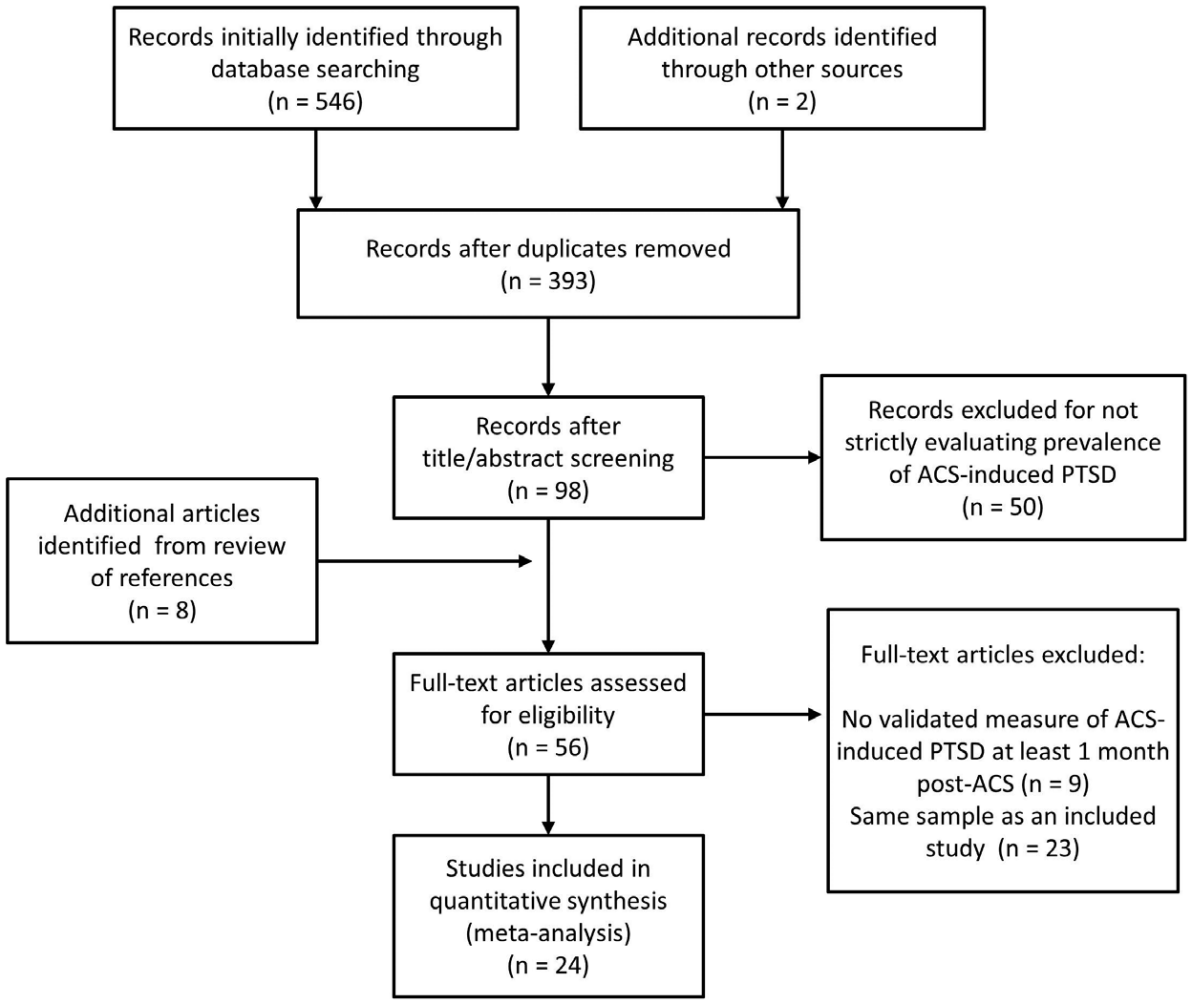
Search strategy flowchart.

We initially identified 548 articles, and 2 coders agreed that 48 articles required full reading. Of these 48 potentially relevant articles plus another 8 identified from references, 24 met our criteria for inclusion (see **Figure 1**).

### Prevalence

**Figure 2:**
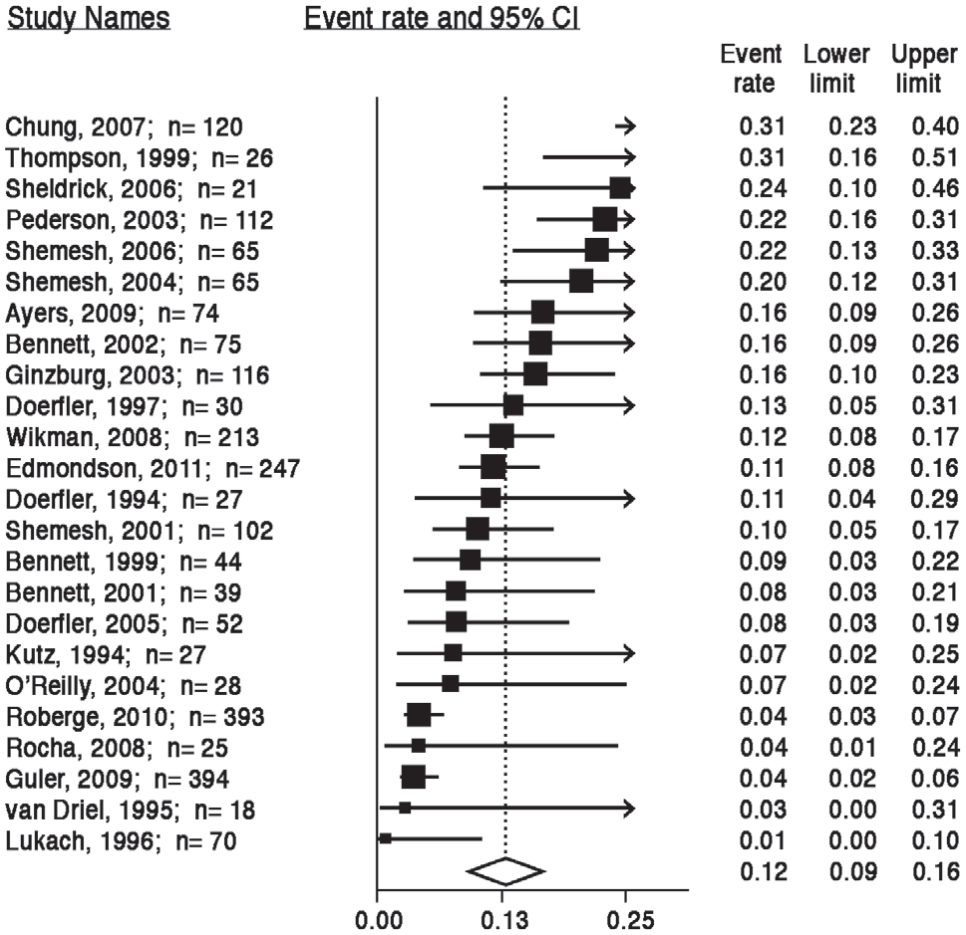
ACS-induced PTSD prevalence estimates. Note: The area of each square is proportional to the study’s weight in the meta-analysis, and each line represents the confidence interval around the estimate. The diamond represents the aggregate estimate, and its lateral points indicate confidence intervals for this estimate.

**Figure 3:**
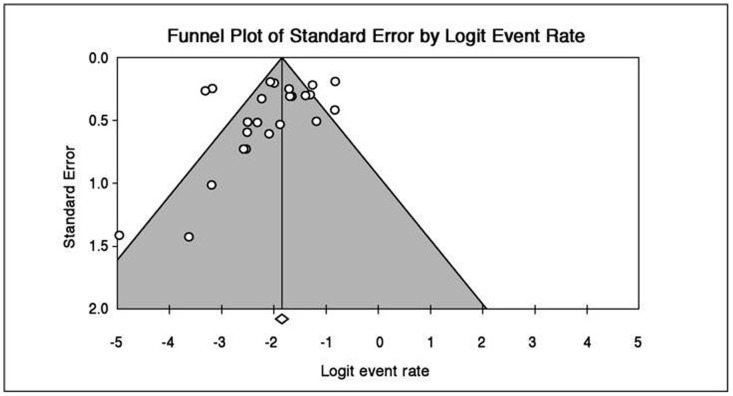
Funnel plot to assess publication bias across prevalence studies.

**Figure 2** shows the prevalence rates of clinically significant symptoms of ACS-induced PTSD reported for each of the 24 studies (N = 2383) and an aggregated estimate of approximately 12% (95% CI, 9%–16%), determined using meta-analytic tests. Significant heterogeneity in prevalence rates existed, with reported rates ranging from 0% to 32% (*Q*_23_ = 120.09; *P*<.001; *I* ^2^ = 80.85). There was no evidence of publication bias (**Figure 3**).

### Sources of Heterogeneity in Prevalence Estimates

#### Assessment method

**Figure 4:**
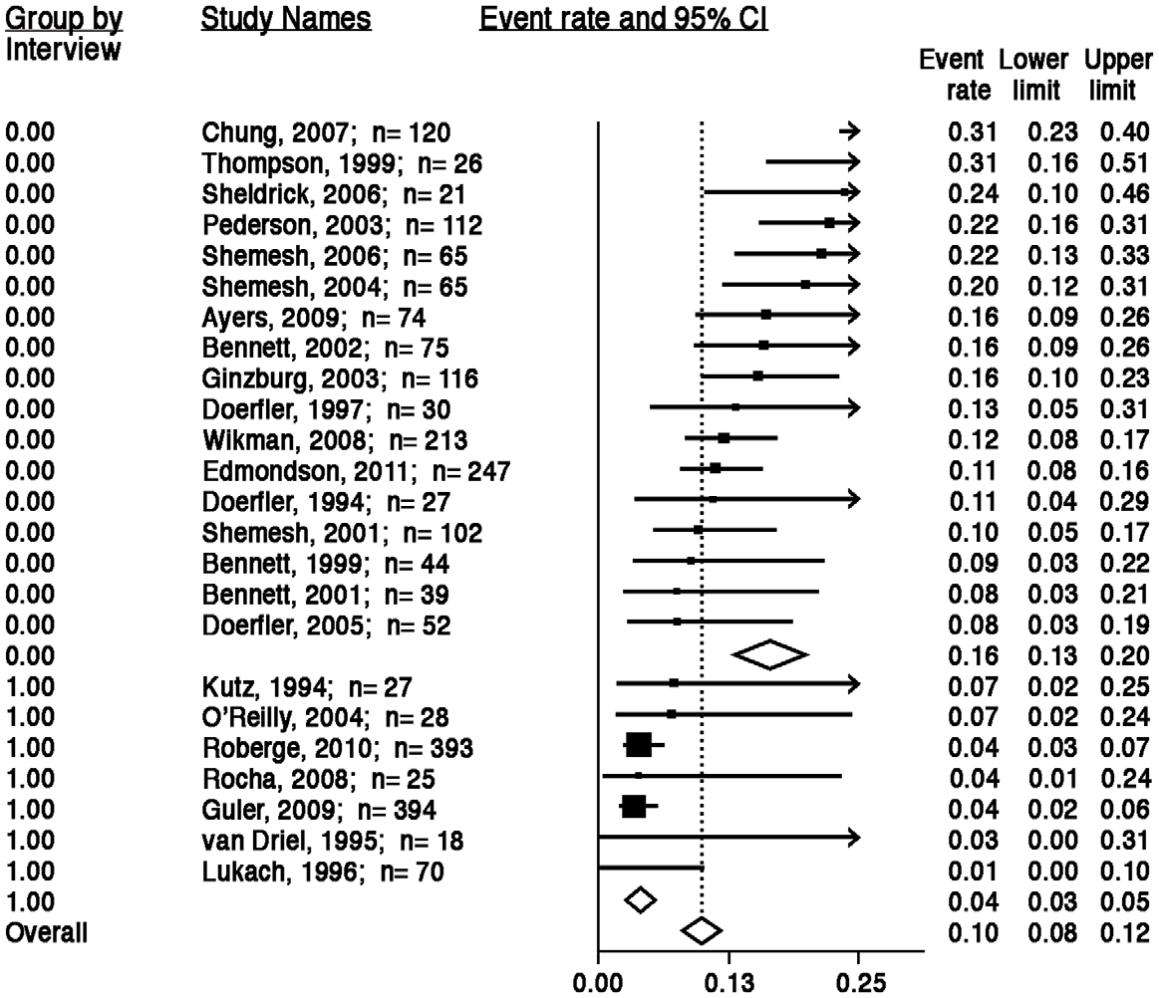
ACS-induced PTSD prevalence estimates by diagnostic interview or screening questionnaire. Note: The area of each square is proportional to the study’s weight in the meta-analysis, and each line represents the confidence interval around the estimate. The diamond represents the aggregate estimate, and its lateral points indicate confidence intervals for this estimate.

The PTSD prevalence rates varied significantly by type of assessment (*Q*_1_ = 59.85; *P*<.001), as shown in **Figure 4**. Compared with the overall aggregate PTSD rate of approximately 12%, the aggregate prevalence estimate in the 17 studies in which patients were assessed solely with screening questionnaires was 16% (95% CI, 13%–20%; *Q*_16_ = 43.89; *P*<.001; *I* ^2^ = 63.55), compared with 4% in the 7 studies in which patients were assessed with clinical interviews (95% CI, 3%–5%; *Q*_6_ = 3.34; *P* = .77; *I* ^2^ = 0.00).

#### Timing of PTSD assessment

The mean number of months between the event and PTSD assessment across studies was 10.24; however, 1 study [13] assessed PTSD almost 10 years after the ACS event and found a prevalence of 31%. Excluding that study, all other studies assessed PTSD within 2 years, with a mean of 5.7 months after ACS. Once the outlier study was excluded, the timing of PTSD assessment was unrelated to prevalence (*P* = .29).

#### Publication date

Later publication date was related to lower estimates of PTSD prevalence and accounted for 5% of the variance in estimates (*P* = .01).

#### Location of study

We compared prevalence rates by whether studies were conducted in the United States (7 studies) [14], [15], [16], [17], [18], [19], [20] or elsewhere (17 studies: Canada [21], Israel [9], [22], [23], [24], United Kingdom [13], [25], [26], [27], [28], [29], [30], [31], [32], and Europe [11], [33], [34]). Study prevalence rates did not vary by study location (*Q*_1_ = .26; *P* = .61).

### Sample Demographics

The mean age of the participants varied from 53 to 67 years. A younger mean age was significantly related to a greater prevalence rate in the 21 studies in which data on age were reported (standardized meta-regression weight  = .25; *P*<.001). The proportion of the study sample that was male varied from 56% to 100% but was unrelated to PTSD prevalence in the 21 studies in which data on sex were reported (*P* = .87). There were not enough studies with racial/ethnic diversity to conduct meta-analytic tests.

#### Inclusion of patients with NSTEMI and UA

Two studies explicitly included patients with NSTEMI or UA (N = 460; prevalence  = 12%; 95% CI = 9%–15%), and their aggregated prevalence estimate did not differ significantly from those of studies that did not explicitly include NSTEMI or UA patients (*P* = .73).

### Association with Clinical Outcomes

**Table 2** provides a description of the 3 prospective studies (N = 609) that reported associations between ACS-induced PTSD and clinical outcomes [9], [10], [17]. Each study used a different PTSD assessment tool; mean patient follow-up varied from 1 to 3.5 years; and sample sizes varied from 65 to 297. The primary clinical outcome for 2 studies [9], [10] was readmission to the hospital for nonfatal cardiac events (i.e., recurrent MI, elective and non elective intracoronary stenting, bypass surgery, pacemaker implantation, cardiac dysrhythmia, cerebrovascular event; Shemesh et al. included hospitalization for hypertension or its complications), and for the third [8] it was a composite of cardiac readmission [i.e., recurrent MI, unstable angina, or urgent/emergency coronary revascularization procedures (percutaneous coronary intervention, coronary artery bypass grafting, or percutaneous transluminal coronary angioplasty)] and all-cause mortality.

**Table 2:** Characteristics of Studies That Estimated an Association Between ACS-Induced PTSD and Adverse Clinical Outcome.

Source, y	N	PTSD, %	Mean Follow-up, y	Study Location	Unstable Angina, Y/N	Clinical Outcome	PTSD Assessmentfor Prediction	Magnitude of Association	Covariates Included in Final Model
Edmondson et al,^24^ 2011	247	11	2	United States	Y	MACE/ACM	IES-R score >33	HR, 1.4 (95% CI,.54–3.46)	Age, sex, Charlson comorbidity index, GRACE, LVEF, BDI
Shemesh et al,^21^ 2004	65	20	1	Israel	N	Cardiac re-hospitalization	IES response consistent with *DSM-IV*	OR, 2.7 (95% CI,1.2–8.8)	none
von Känel et al,^22^ 2011	213	12	2.8	Switzerland	Y	MACE	Per 10 points on PDS	HR, 1.4(95% CI,1.07–1.88)	Age, sex, hypertension

Abbreviations: ACM, all-cause mortality; ACS, acute coronary syndrome; BDI, Beck Depression Inventory; CI, confidence interval; *DSM-IV, Diagnostic and Statistical Manual of Mental Disorders (Fourth Edition);* IES, Impact of Events Scale [64]; IES-R, Impact of Events Scale–Revised [65]; GRACE, Global Registry of Acute Cardiac Events; LVEF, left ventricular ejection fraction; MACE, major adverse cardiac events; PDS, Posttraumatic Stress Diagnostic Scale [66]; PTSD, posttraumatic stress disorder.

**Figure 5:**
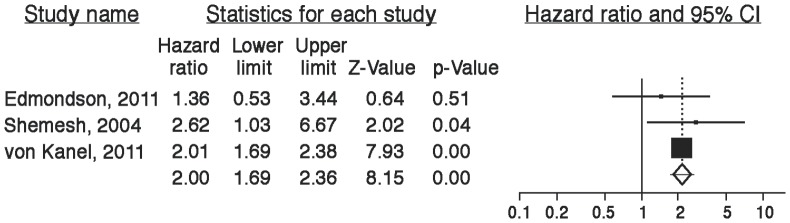
Association of ACS-induced PTSD with adverse clinical outcome. Note: The size of the box associated with each study’s estimate represents the precision of the estimate, and the line represents the confidence interval around the estimate.

**Figure 6:**
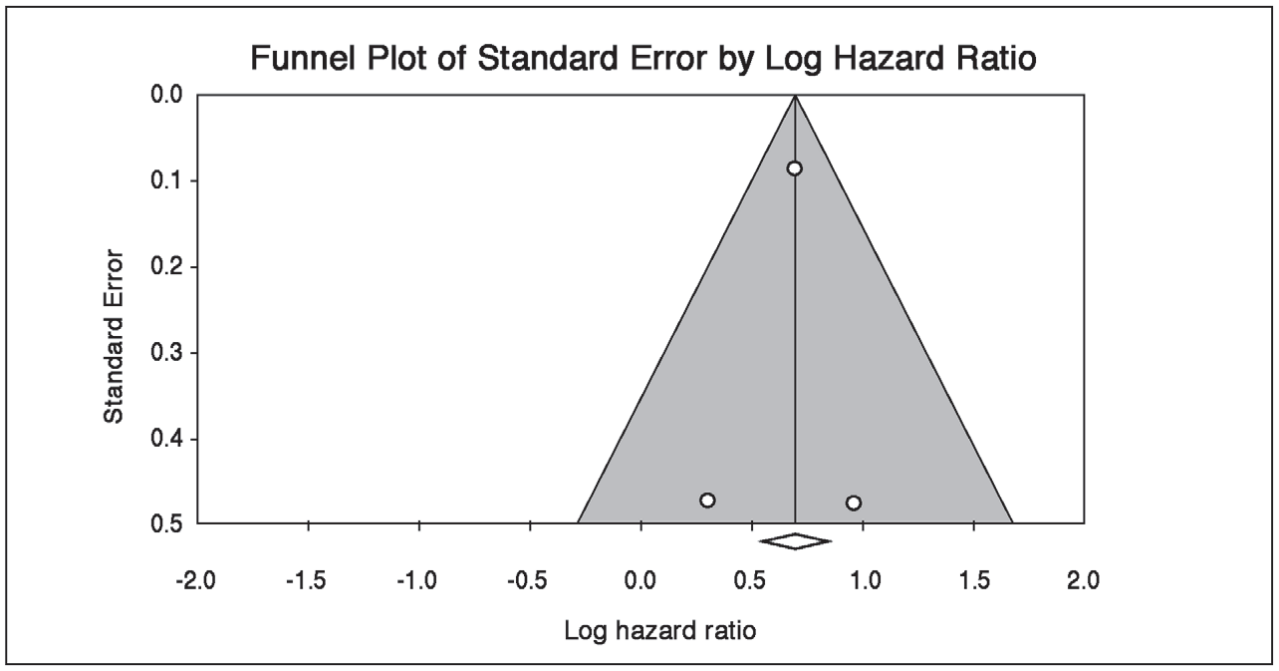
Funnel plot to assess publication bias across outcome studies.

Using the most conservative estimates available (**Figure 5**), the risk ratio associated with a positive screen for ACS-induced PTSD was 2.00 (95% CI, 1.69–2.37). While publication bias is difficult to assess with 3 studies, examination of the funnel plot did not suggest missing studies in which no relationship between PTSD symptoms and clinical outcomes was found (**Figure 6**); a fail-safe N estimate suggested that 27 such null studies would be required for the aggregate point estimate to be nonsignificant. We conducted a sensitivity analysis by calculating the aggregate risk ratio without the Shemesh et al. study because it was not powered to adjust for covariates that may have attenuated the association between ACS-induced PTSD and clinical outcomes. The aggregate risk ratio without the Shemesh et al. study was only slightly smaller (risk ratio, 1.98; 95% CI, 1.68, 2.35), suggesting that the main finding was not unduly influenced by the Shemesh et al. study.

## Discussion

In recent years, cardiologists and the broader medical community have become increasingly aware that psychological disorders, particularly depression, are common in patients with ACS and are associated with adverse clinical outcomes [35], [36], [37], [38], [39]. Even so, abundant evidence suggests that psychological disorders are underrecognized and undertreated in cardiac populations [40], [41]. While awareness of depression has increased in cardiology practice, awareness of the possibility of PTSD due to ACS has lagged, possibly because many still see PTSD as primarily a disorder of combat veterans or sexual assault survivors. Indeed, even in academic and community mental health clinics, only a very small proportion of PTSD cases from all causes are identified [42], [43]. This under-diagnosis is likely more pronounced in cardiology practice.

To our knowledge, this is the first meta-analytic review of ACS-induced PTSD. We found a 12% prevalence of clinically significant ACS-induced PTSD among ACS patients and, based on a small number of studies, a doubling of risk of mortality and recurrent cardiac events among ACS patients with PTSD symptoms. In previous qualitative literature reviews, [44], [45] investigators have examined the possible risk for PTSD following ACS. However, those reviews do not include more recent, larger, and methodologically rigorous studies that investigated both prevalence of ACS-induced PTSD and its association with risk of ACS recurrence and mortality. Given that 1.4 million patients are discharged from US hospitals each year with a diagnosis of ACS [46], these results suggest that 168 000 ACS patients in the United States alone will develop clinically significant PTSD symptoms due to ACS. Among ACS patients in the United States, 20% are hospitalized again within 1 year of the ACS event [47], and 60% of the $1.5 billion spent annually to treat ACS in the United States is due to subsequent hospitalizations [47]. It appears that ACS-induced PTSD may contribute to repeated hospitalization and mortality at a rate very similar to that reported for depression (approximate doubling of risk [48]).

### Prevalence of ACS-Induced PTSD

Although the overall prevalence of ACS-induced PTSD was 12%, individual study prevalence estimates ranged from 0% to 32%, suggesting a considerable degree of heterogeneity. As expected, prevalence estimates based on screening questionnaires were greater than estimates based on clinical interviews. Also, similar to within-study findings that younger age is associated with greater likelihood of ACS-induced PTSD, younger mean sample age was related to greater prevalence estimates across studies. Finally, later publication date was associated with lower prevalence rates, though the association was modest, which may reflect reduced psychological trauma due to advances in treatment or the fact that the definition of ACS has broadened in recent years to include less severe disease. However, since we found that the inclusion of patients with less severe ACS (ie, NSTEMI and UA) was unrelated to prevalence estimates, advances in treatment may well be responsible for slightly better post-ACS psychological outcomes in recent years. Timing of PTSD assessment, study location, and sex of the sample were unrelated to prevalence estimates.

### Symptoms and Clinical Outcomes of ACS-Induced PTSD

Our analyses indicated that ACS-induced PTSD symptoms are associated with an approximate doubling of risk for recurrent cardiac events or mortality. Two of the 3 studies included in the analysis [10], [17] adjusted for multiple demographic and clinical covariates, suggesting that the association is not likely to be confounded by demographic characteristics and disease severity.

Important caveats to this review include the fact that only studies with relatively small sample sizes have addressed ACS-induced PTSD, many of which were marginally powered to detect associations with outcomes over relatively short intervals. Furthermore, no study to date has been sufficiently powered to test for potential mechanisms or effect modifiers of the relationship between ACS-induced PTSD and adverse clinical outcomes. Thus, although this meta-analysis was able to quantify more precisely the magnitude of the increased risk of clinical events due to ACS-induced PTSD symptoms, a clear need for additional research remains.

Caveats aside, there is now considerable evidence for a link between PTSD due to various life stressors and subsequent heart disease [49], but the mechanisms for this association are not yet known [50]. If the apparent association between ACS-induced PTSD and ACS recurrence and mortality holds up to further scrutiny, many of the candidate mechanisms that require years to develop and be associated with an increase in risk, and so more proximate or trigger mechanisms should be explored, as the follow-up between PTSD and a recurrence/mortality is fairly short in this review (ie, follow-up periods for the 3 included studies ranged from 1–3 years). We know that both ACS and PTSD are associated with sympathetic activation and elevated proinflammatory cytokines, including C-reactive protein [51], tumor necrosis factor, and interleukin 1 [51]. It is likely that the additive effects of the inflammation found in patients with PTSD may adversely affect the heart [52]. Pharmacologic and psychotherapy treatments for PTSD due to noncardiac events reduce PTSD symptoms [53] and might reduce accompanying inflammation, potentially producing more favorable clinical outcomes in ACS patients. It seems likely, but has not yet been tested, that reducing symptoms of PTSD could improve adherence to post-ACS treatment regimens [9], [54].

### Study Limitations

The present review has several limitations. First, since we did not include unpublished articles or articles from non–peer-reviewed journals, we are more likely to have excluded negative findings [55]. Second, although some articles included in this review [17], [56] were from samples large enough to report useful data on within-sex, race, or ethnicity estimates, or rates by ACS type, our findings were based on cruder estimates, such as the proportion of each sample that was male, which may limit our conclusions about the relationship of demographic factors to prevalence. Finally, none of the studies included in this review reported on the severity of the ACS event in terms of the types of critical care that may have become necessary after the ACS, such as intensive care unit (ICU) admission or major surgery. While there seemed to be no difference in PTSD rates by ACS type (STEMI vs NSTEMI/UA), a growing literature suggests that exposure to critical care for other types of medical events can induce PTSD in up to 64% of ICU patients [57].

### Future Directions

We were able to locate only one PTSD treatment study in ACS patients: a phase I safety trial of cognitive behavioral therapy for ACS-induced PTSD [58]. The study was not powered to detect differences between the treatment and control groups (the intent-to-treat analysis included 51 participants), but small-to-moderate reductions in PTSD symptoms were reported, and no adverse clinical outcomes or safety concerns were associated with treatment. Thus, more research is needed to determine whether treatment can reduce ACS-induced PTSD symptoms and reduce the associated risk for ACS recurrence and mortality.

While little research exists on treatment of ACS-induced PTSD, a growing body of literature suggests risk factors for PTSD diagnosis and symptom severity after ACS. These risk factors include intense fear [28], perceived life threat, lack of control [59], helplessness and chest pain [60] during the ACS, dissociation during the event, acute stress disorder [41], depression symptoms during hospitalization [21], and history of psychiatric disorder before ACS [21], alexithymia [26], or neuroticism [33]. Demographic factors associated with ACS-induced PTSD in some studies include younger age [61], female sex [21], ethnic minority status, and low socioeconomic status [61]. While these scattered risk factor studies have been useful, a unified risk stratification strategy is warranted for predicting which patients are most likely to develop ACS-induced PTSD.

In conclusion, across published studies concerning the prevalence of ACS-induced PTSD and its associations with clinical outcomes, a few general conclusions can be drawn: (1) ACS is a traumatic event for many, and ACS-induced PTSD is relatively common, with approximately 12% experiencing clinically significant PTSD symptoms and 4% meeting full diagnostic criteria for the disorder, and (2) based on a small number of studies, clinically significant symptoms of ACS-induced PTSD appear to double patients’ risk of recurrent cardiac events and mortality. These results identify areas requiring further research; in particular, studies testing treatments for ACS-induced PTSD are needed.
