# Correction: Posttraumatic Stress Disorder Prevalence and Risk of Recurrence in Acute Coronary Syndrome Patients: A Meta-analytic Review

**DOI:** 10.1371/journal.pone.0213635

**Published:** 2019-03-06

**Authors:** Donald Edmondson, Safiya Richardson, Louise Falzon, Karina W. Davidson, Mary Alice Mills, Yuval Neria

There are a number of errors in the citations in Table 1. Please see the correct Table 1 here.

**Table 1:** Characteristics of Studies on the Prevalence of ACS-Induced PTSD.

Source, y	PTSD Prevalence, %	PTSD Measure	Clinical Interview, Y/N	Time After ACS Event, mo	Study Location	N	Male, %	Mean Age, y	Included Unstable Angina, Y/N
Ayers et al,^25^ 2009	16	PDS	N	2	United Kingdom	74	76	62	N
Bennett and Brooke,^26^ 1999	11	PDS	N	9.24	United Kingdom	44	68	62.5	N
Bennett et al,^27^ 2001	8	PDS	N	3	United Kingdom	39	77	59.7^a^	N
Bennett et al,^28^ 2002	16	PDS	N	3	United Kingdom	75	78^a^	60.4^a^	N
Chung et al,^13^ 2007	31	PDS	N	115.8	United Kingdom	120	78	66.85	N
Doerfler et al,^16^ 1994	11	Multiple	N	9	United States	27	100	59.1	N
Doerfler,^14^ 1997	11	PDS	N	1	United States	30	71^a^	58.9	N
Doerfler et al,^15^ 2005	8	PSS-SR	N	4.5	United States	52	69	57.73	N
Edmondson et al,^8^ 2011	11	IES-R	N	1	United States	247	55	60	Y
Ginzburg et al,^22^ 2003	16	PTSD-I	N	7	Israel	116	81	54.95	N
Guler et al,^11^ 2009	4	CAPS	Y	3.25	Switzerland	394	83	61	N
Kutz et al,^29^ 1994	7	SCID	Y	9.6	United Kingdom	27	89	58.2	N
Lukach,^18^ 1996	0	SCID	Y	6	United States	70	69	59.3	N
O'Reilly et al,^30^ 2004	7	SCID	Y	10.7	United Kingdom	28	89	58.2	N
Pedersen et al,^33^ 2003	22	PDS	N	1.25	Denmark	112	71^a^	60	N
Roberge et al,^21^ 2010	4	SCID	Y	1	Canada	393	NR	59.22	N
Rocha et al,^19^ 2008	4	SCID	Y	1.5	United States	25	NR	NR	N
Sheldrick et al,^31^ 2006	24	DTS	N	1.5	United Kingdom	21	NR	NR	N
Shemesh et al,^24^ 2001	10	IES	N	6	Israel	102	79	61.3	N
Shemesh et al,^9^ 2004	20	IES	N	6	Israel	65	79^a^	53^a^	N
Shemesh et al,^23^ 2006	22	IES	N	7.5	Israel	65	90	58	N
Thompson,^20^ 1999	31	ADIS-IV	N	6	United States	26	NR	NR	N
van Driel et al,^34^ 1995	0	SCID	Y	24	Holland	18	56	61.96^a^	N
Wikman et al,^32^ 2008	12	PSS-SR	N	12	United Kingdom	213	77	61	Y

Abbreviations: ACS, acute coronary syndrome; ADIS-IV, Anxiety Disorder Interview Schedule for *DSM-IV* [3]; CAPS, Clinician Administered PTSD Scale [62]; DTS, Davidson Trauma Scale [63]; IES, Impact of Events Scale [64]; IES-R, Impact of Events Scale–Revised [65]; NR, not reported; PDS, Posttraumatic Stress Diagnostic Scale [66]; PSS-SR, PTSD Symptom Scale–Self-Report Version [67]; PTSD, posttraumatic stress disorder; RI, Reaction Index [68]; SCID, Structured Clinical Interview for *DSM-IV* [69];

^a^ Value reported for full sample, including participants who did not complete the study.
